# Effectiveness of a multimodal hand hygiene campaign and obstacles to success in Addis Ababa, Ethiopia

**DOI:** 10.1186/2047-2994-3-8

**Published:** 2014-03-17

**Authors:** Karen Schmitz, Russell R Kempker, Admasu Tenna, Edward Stenehjem, Engida Abebe, Lia Tadesse, Ermias Kacha Jirru, Henry M Blumberg

**Affiliations:** 1 Emory University School of Medicine, 1648 Pierce Drive NE, Atlanta, GA 30322, USA; 2 Division of Infectious Diseases, Department of Medicine, Emory University School of Medicine, 49 Jesse Hill Jr Drive, Atlanta, GA 30303, USA; 3 Division of Infectious Diseases, Department of Medicine, Addis Ababa University, P.O. Box 24792 Code 1000, Addis Ababa, Ethiopia; 4 Department of Clinical Epidemiology and Infectious Diseases, Intermountain Medical Center, 5212 Cottonwood Street, LL2, Murray, UT 84157, USA; 5 Department of Surgery, St. Paul’s Hospital Millennium Medical College, P.O. Box 1271, Addis Ababa, Ethiopia; 6 Department of Obstetrics and Gynecology, St. Paul’s Hospital Millennium Medical College, P.O. Box 1271, Addis Ababa, Ethiopia; 7 Departments of Epidemiology and Global Health, Emory Rollins School of Public Health, 1518 Clifton Rd NE, Atlanta, GA 30329, USA; 8 120 S. Pennsylvania St #101, Denver, CO 80209, USA

**Keywords:** Hand hygiene, Ethiopia, World Health Organization, Infection control, Health personnel

## Abstract

**Background:**

Hand hygiene is the cornerstone of infection control and reduces rates of healthcare associated infection. There are limited data evaluating hand hygiene adherence and hand hygiene campaign effect in resource-limited settings, especially in Sub-Saharan Africa. This study assessed the impact of implementing a World Health Organization (WHO)-recommended multimodal hand hygiene campaign at a hospital in Ethiopia.

**Methods:**

This study included a before-and-after assessment of health care worker (HCW) adherence with WHO hand hygiene guidelines. It was implemented in three phases: 1) baseline evaluation of hand hygiene adherence and hospital infrastructure; 2) intervention (distribution of commercial hand sanitizer and implementation of an abbreviated WHO-recommended multimodal hand hygiene campaign); and 3) post-intervention evaluation of HCW hand hygiene adherence. HCWs’ perceptions of the campaign and hand sanitizer tolerability were assessed through a survey performed in the post-intervention period.

**Results:**

At baseline, hand washing materials were infrequently available, with only 20% of sinks having hand-washing materials. There was a significant increase in hand hygiene adherence among HCWs following implementation of a WHO multimodal hand hygiene program. Adherence increased from 2.1% at baseline (21 hand hygiene actions/1000 opportunities for hand hygiene) to 12.7% (127 hand hygiene actions /1000 opportunities for hand hygiene) after the implementation of the hand hygiene campaign (OR = 6.8, 95% CI 4.2-10.9). Hand hygiene rates significantly increased among all HCW types except attending physicians. Independent predictors of HCW hand hygiene compliance included performing hand hygiene in the post-intervention period (aOR = 5.7, 95% CI 3.5-9.3), in the emergency department (aOR = 4.9, 95% CI 2.8-8.6), during patient care that did not involve Attending Physician Rounds (aOR = 2.4, 95% CI 1.2-4.5), and after patient contact (aOR = 2.1, 95% CI 1.4-3.3). In the perceptions survey, 64.0% of HCWs indicated preference for commercially manufactured hand sanitizer and 71.4% indicated their hand hygiene adherence would improve with commercial hand sanitizer.

**Conclusions:**

There was a significant increase in hand hygiene adherence among Ethiopian HCWs following the implementation of a WHO-recommended multimodal hand hygiene campaign. Dissatisfaction with the current WHO-formulation for hand sanitizer was identified as a barrier to hand hygiene adherence in our setting.

## Background

Hand hygiene has long been regarded as the cornerstone of infection control efforts and an essential measure for prevention of healthcare-associated infections (HCAIs) [1]. Despite the importance of hand hygiene in the healthcare setting, adherence to hand hygiene standards remains universally low. In the United States, rates of adherence have been shown to be as low as 36% but there has been substantial attention paid to increasing adherence based on patient safety concerns and regulatory and accreditation agency requirements [2]. Limited data from low and middle-income countries suggest that hand hygiene adherence rates are very low in resource limited areas, with baseline reports as low as 5% of all opportunities for hand hygiene [3].

Numerous strategies have been evaluated in an attempt to improve rates of hand hygiene, including those that focus on infrastructure changes, education, visual reminders, or ongoing monitoring and feedback programs. While these individual components have proven effective, interventions that combine these strategies into multimodal hand hygiene campaigns appear to be the most successful in improving hand hygiene adherence by health care workers (HCWs) [1,4-7]. The World Health Organization (WHO) has adopted an evidence-based multimodal hand hygiene strategy as part of the First Global Patient Safety Challenge [1]. The WHO Multimodal Hand Hygiene Strategy has been implemented extensively in high income, resource intensive countries, however there remains limited data on the impact of such programs in resource-limited countries, especially in Sub-Saharan Africa [8-11].

Effective hand hygiene campaigns are urgently needed in developing countries, where the prevalence of HCAIs is estimated to be at least three times higher than in the USA and Europe [12]. Poor hand hygiene in resource-limited settings likely play a role in nosocomial transmission of bacterial pathogens and are important cause of the high rates of HCAIs. Significant cultural, behavioral, and institutional factors have been identified as unique barriers to appropriate hand hygiene adherence in these settings [1].

While rates of HCAIs are incompletely defined in Ethiopia, they are assumed to be high; one recent study reported nosocomial infections in 39% of hospitalized patients [13]. To combat presumed high rates of HCAIs, the Ethiopian Federal Ministry of Health has prioritized the implementation of several hospital infection control measures, including hand hygiene [14]. Despite this emphasis by the Ministry of Health, there are no prior reports or assessments on how well infection control practices have been implemented. No studies have been done to assess hand hygiene adherence in Ethiopia, and there remains a paucity of data evaluating the impact of the WHO Multimodal Hand Hygiene Strategy in Sub-Saharan Africa [1,11]. The purpose of this study was to define baseline rates of HCW hand hygiene adherence and assess the impact of implementing the WHO Multimodal Hand Hygiene Strategy at an academic hospital in Addis Ababa, Ethiopia.

## Methods

This study took place at a 278 bed university-affiliated teaching hospital in in Addis Ababa, Ethiopia from May 7, 2012 to August 10, 2012. Hospital leadership was involved in project conception, design and implementation. The project was conducted in collaboration with the hospital’s Infection Control Department. The project was approved by the Institutional Review Boards (IRBs) at Emory University and St. Paul’s Millennium Medical College.

### Phase 1: Baseline evaluation

The baseline evaluation phase took place over 4 weeks and included a series of direct witnessed observations of HCW hand hygiene practices. All categories of HCWs with direct patient contact were eligible for observation. All hand hygiene observations were performed by a single observer (KS), who was trained in accordance with the WHO’s hand hygiene observation method [15]. Observations took place in all inpatient hospital wards with the exception of the pediatrics ward, which was excluded due to the small size of the ward and logistical issues including the inability to unobtrusively observe HCWs hand hygiene practices on this ward. Observations were exclusively performed during day shift for similar logistical reasons.

Opportunities for hand hygiene were adapted from the WHO’s “patient zone and health-care area” model for hand hygiene, and included (1) before patient contact and (2) after contact with a patient or patient surroundings. The 5 moments of hand hygiene [1] were not employed in this study due to the complexity of the model and the relative infrequency of aseptic procedures in this setting. Adherence was defined as use of waterless hand sanitizer or soap and water during any instances of the above indications. Adherence was calculated by the number of times hand hygiene was performed, divided by the number of total opportunities for hand hygiene (Adherence = number of hand hygiene actions/number of opportunities for hand hygiene). The following data were recorded for each observation: date, location, professional category, indication, and if the encounter occurred on attending physician rounds (Additional file 1).

A one-time facilities assessment was performed as part of the baseline evaluation. Data were collected from all inpatient hospital wards as well as from the Emergency Department. An inventory of hand hygiene resources was modified from the WHO Ward Infrastructure Survey [16] and included information on number and functionality of sinks, the number of beds, and the availability of soap, alcohol based hand sanitizer, and drying materials.

### Phase 2: Intervention

The intervention phase took place over a 6 week period and included the implementation of an abbreviated version of the WHO Multimodal Hand Hygiene Strategy [1]. The campaign utilized a five-component approach to increase HCW hand hygiene adherence. The first component consisted of infrastructure change, which was accomplished by making soap and commercially prepared waterless hand sanitizer available, which had not previously been available in the hospital. The new commercial sanitizer was purchased from Purell, GOJO Industries, Akron, OH, USA by the study institution at a cost of approximately 1.33 USD per 112 ml bottle, for a total cost of 665 USD per month. All HCWs, including those HCWs in wards excluded from observation (e.g. night shift workers and those in pediatrics wards) received a small bottle (112 ml) of hand sanitizer for their personal use while in the hospital; refills of were available throughout the intervention and post-intervention phases. Soap was made available at all sinks throughout the hospital throughout the intervention and post-intervention period, to ensure that all HCWs had access to hand hygiene materials for the duration of the intervention and post-intervention period. All hand hygiene products that existed prior to the intervention (soap and locally produced sanitizer) were left in place for the duration of the intervention and post-intervention periods.

The second component consisted of training and education of HCWs. The initial training took place over a 6-week period and took the form of a 30 minute didactic education session that focused on the importance of hand hygiene in improving patient safety and quality of care for patients. Training materials were adapted from WHO hand hygiene training guides [17]. Presentations were given by Ethiopian physicians and were given in both English and Amharic. Training sessions were made available to all HCWs, including those not included in study observations (e.g. night shift workers and those in pediatrics wards). Post-intervention training occurred on an ongoing basis and included informal teaching sessions given by members of the hand hygiene committee during rounds. The third component consisted of posting visual reminders for hand hygiene throughout the entire hospital. Over 250 posters were displayed throughout the hospital (Additional file 2). The fourth component was development of Institutional Safety Climate, which was accomplished by the development of “hand hygiene champions” who were leaders at the hospital and served as role models and facilitators of change. Hand Hygiene Champions were primarily nurse leaders, but also included sanitation workers, physicians, and the Dean of Students. The fifth component consisted of monitoring and providing feedback on hand hygiene practices. The nurse hand hygiene champions were trained in accordance with the WHO hand hygiene observation method [15] and performed weekly observations of their designated wards and provide feedback to HCWs on hand hygiene adherence and provided encouragement to HCWs in their areas.

### Phase 3: Post-intervention evaluation

The follow up evaluation phase took place over a 4-week period, immediately following the intervention phase. During the post-intervention evaluation, additional hand hygiene observations were made to determine if hand hygiene adherence improved following the intervention period. These follow up HCW hand hygiene observations were carried out in the same wards as phase 1, using the same methodology, and by the same observer (KS) that conducted the Phase 1 (baseline) evaluations.

A final component of this study included a self-completed perceptions survey of HCWs at the teaching hospital in Addis Ababa. The survey focused on HCW acceptance of and attitudes toward the hand hygiene campaign, current hand hygiene practices, and their perceptions of the hand sanitizer (both the commercially manufactured product and the product prepared by the hospital on site using the WHO-recommended formulation). All HCWs at the hospital were eligible for the survey. The questionnaire included 26 questions and included both WHO developed items [18] and internally developed items (Additional file 3).

### Data analysis

In order to assess HCW hand hygiene practices before and after our intervention, we sought to include 2,000 witnessed opportunities for hand hygiene (1000 at baseline and 1000 in the post-intervention post-intervention phase). The relatively large sample allowed observations in multiple departments and the ability to include multiple types of health care workers in the observations. Data from hand hygiene observations were collected on paper forms, which were subsequently entered into an Excel database. Data Analyses were performed using SAS, version 9.3 (SAS Inc., Cary, NC). Hand hygiene adherence is expressed as the proportion of predefined opportunities met by hand hygiene actions. A χ^2^ statistic was used to compare rates of baseline and post-intervention hand hygiene adherence rates overall and among different groups. Univariate and multivariate logistic regression analysis was used to evaluate the association of individual predictors of hand hygiene adherence. The primary predictor variable was period of observation (pre or post intervention). Other predictors and potential confounders included in the logistic regression analyses were location of hand hygiene observation, type of HCW (e.g., faculty physician, resident physician, medical student, nurse, etc.), and indication (before vs. after patient contact) observation. A p-value <0.05 was considered to be statistically significant.

Data from the perceptions survey completed by HCWs were entered into an online REDCap database [19] and descriptive statistics were used to report survey results.

## Results

### Infrastructure

The results of the baseline facilities assessment are shown in Table 1. All 11 patient wards had functioning sinks, with a sink to patient bed ratio of 1 sink to 4.6 patient beds. However, hand-washing materials were infrequently available with only 20% of sinks having soap and none of the sinks had drying materials (such a cloth towel or paper towels). Waterless hand sanitizer (made by the hospital in accordance with the WHO-recommended formulation) [5] was available in only 36% of wards at baseline prior to the intervention.

**Table 1:** Baseline hand hygiene infrastructure/resources

**Characteristic**	**N (%)**
Total beds	278
Total wards	11
Wards with sinks	11 (100.0%)
Wards with hand sanitizer	4 (36.4%)
Total sinks	60
Functioning sinks	60 (100.0%)
Sinks with soap available	12 (20.0%)
Sink to patient bed ratio	1:4.6

### Health care worker hand hygiene adherence

A total of 2000 opportunities for hand hygiene were observed during the study, (1000 during the baseline assessment period and 1000 in the post-intervention period). The observations were evenly divided between baseline and post-intervention phases and amongst 4 hospital wards: Internal Medicine, Surgery, Obstetrics and Gynecology, and Emergency Room (Table 2).

**Table 2:** Health care worker hand hygiene adherence rates at baseline and following implementation of the WHO multimodal hand hygiene strategy

**Characteristic**	**Hand hygiene adherence**		
	**[Number adherent/number of observations (%)]**		
	**Baseline**	**Post-intervention**	***P***^**c**^
**Total**	21/1000 (2.1%)	127/1000 (12.7%)	<0.001
**Location of observation**			
Emergency dept.	11/217 (5.1%)	62/250 (24.8%)	<0.001
Medicine ward	4/265 (1.5%)	25/250 (10.0%)	<0.001
OB/GYN^a^ ward	3/200 (1.5%)	23/250 (9.2%)	<0.001
Surgery ward	3/318 (0.9%)	17/250 (6.8%)	<0.001
**Type of HCW**			
Attending	2/69 (2.9%)	4/69 (5.8%)	0.40
Resident	7/436 (1.6%)	30/211 (14.2%)	<0.001
Medical student	3/291 (1.0%)	20/254 (7.9%)	<0.001
Nurse	5/144 (3.5%)	62/322 (19.3%)	<0.001
Other HCWs^b^	4/60 (6.7%)	11/144 (7.6%)	0.81
**Timing**			
During attending rounds	5/351 (1.4%)	9/205 (4.4%)	0.03
Not during rounds	16/649 (2.5%)	118/795 (14.8%)	<0.001
**Patient contact**			
Before patient contact	3/398 (0.8%)	25/250 (10.0%)	<0.001
After patient contact	18/602 (3.0%)	102/750 (13.6%)	<0.001

^a^OB/GYN: obstetrics and gynecology; ^b^HCW: health care worker; ^c^ P-value between pre and post intervention period.

Hand hygiene adherence rates increased significantly from 2.1% (21/1000) at baseline to 12.7% (127/1000) following the implementation of the WHO Multimodal Hand Hygiene Strategy (OR = 6.8, 95% CI 4.2-10.9, p < 0.001). Hand hygiene rates increased significantly from the baseline period to the post-intervention period among all HCW types except for attending physicians (Table 2). Nurses had better hand hygiene adherence when compared with the reference group (aOR = 3.7, 95% CI 1.6-8.7, p = 0.003), however this was significant only in the univarite analysis. In multivariate analysis (Table 3), independent predictors of HCW hand hygiene adherence included performing hand hygiene in the post-intervention period (aOR = 5.7, 95% CI 3.5-9.3, p < 0.001), in the emergency department as compared to the surgical ward (aOR = 4.9, 95% CI 2.8-8.6, p < 0.001), during patient care that did not involve Attending Physician Rounds (aOR = 2.4, 95% CI 1.2-4.5, p = 0.009), and after patient contact (aOR = 2.1, 95% CI 1.4-3.3, p = 0.001).

**Table 3:** Univariate and multivariate analysis of predictors for hand hygiene adherence

**Characteristic**	**Univariate analysis**		**Multivariate analysis**	
	**OR (95% CI)**	***P***	**aOR (95% CI)**	**P**^**c**^
Post-intervention period (vs. baseline period)	6.8 (4.2-10.9)	<0.001	5.7 (3.5-9.3)	<0.001
Location of observation				
Surgery ward	1.00	-	1.00	-
Medicine ward	1.6 (0.9-2.9)	0.10	1.8 (1.0-3.3)	0.07
OB/GYN^a^ ward	1.7 (0.9-3.1)	0.09	1.7 (0.9-3.2)	0.11
Emergency dept.	5.1 (3.0-8.5)	<0.001	4.9 (2.8-8.6)	<0.001
Type of HCW^b^				
Attending physician	1.00	-	1.00	-
Resident physician	1.3 (0.6-3.2)	0.52	1.4 (0.6-3.7)	0.46
Medical student	1.0 (0.4-2.4)	0.95	0.50 (0.2-1.4)	0.17
Nurse	3.7 (1.6-8.7)	0.003	1.2 (0.5-3.2)	0.68
Other HCW	1.8 (0.7-4.6)	0.26	0.89 (0.3-2.6)	0.83
Timing of hand hygiene				
During attending rounds	1.00	-	1.00	-
Not during attending rounds	4.0 (2.2-23.2)	<0.001	2.4 (1.2-4.5)	0.009
Patient contact				
Before contact	1.00	-	1.00	-
After contact	2.2 (1.4-3.3)	<0.001	2.1 (1.4-3.3)	0.001

^a^OB/GYN: obstetrics and gynecology; ^b^HCW: health care worker; ^c^P-value between pre and post intervention period.

OR = Odds Ratio; aOR = Adjusted Odds Ratio.

### Healthcare workers knowledge, attitudes and practice

Among 212 HCWs approached, a total of 161 completed the post-intervention perceptions survey (Additional file 3 and Table 4). The mean age of participants was 26 years and 55.9% were female. One hundred thirty (80.7%) of 161 HCWs who completed the perceptions survey had undergone formal hand hygiene training during the intervention period. Of the majority of HCWs that had undergone formal hand hygiene training, 85.4% (111/130), stated that the training increased their knowledge of the importance of hand hygiene for infection control and 80.0% (104/130) agreed or strongly agreed that the training increased their frequency of hand hygiene (Table 5).

**Table 4:** Demographics of health care workers who completed a post-intervention perceptions survey (N = 161)

**Characteristic**	**N (%)**
Mean age, years (IQR)	26 (23–28)
Female	90 (55.9)
Job title	
Physician	28 (17.4)
Medical student	68 (42.2)
Nurse	59 (36.6)
Other	6 (3.7)
Department	
Internal medicine	39 (24.2)
Surgery	38 (23.6)
Obstetrics/Gynecology	40 (24.8)
Pediatrics	13 (8.1)
Emergency medicine	10 (6.2)
Other	21 (13.0)
Currently in possession of commercially made hand sanitizer (distributed as part of hand hygiene campaign)	123 (76.4)

IQR = Interquartile Range.

**Table 5:** **Health care workers’ perceptions of hand hygiene training (N = 130)**^a^

**Characteristic**	**A lot/a great deal**	**Somewhat**	**Not at all/little**
	**N (%)**		
To what extent did training improve your knowledge of the importance of hand hygiene for infection control	111 (85.4)	17 (13.0)	2 (1.5)
To what extent did training improve your understanding of when and how to perform hand hygiene	113 (86.9)	15 (11.5)	2 (1.5)
To what extent did training increase your frequency of hand hygiene	104 (80.0)	23 (17.6)	3 (2.3)

^a^130/161 indicated that they had completed training, all others were excluded from this analysis.

The surveyed HCWs preferred the commercially manufactured sanitizer to the hospital prepared version. HCWs who completed the perceptions survey indicated that the hospital prepared sanitizer was more likely to cause drying of their hands compared to the commercially prepared sanitizer (76/161 [47.2%] vs. 21/161 [13.0%], OR = 6.0, 95% CI 3.4-10.4) (Table 6). 64.0% of HCWs indicated that they prefer commercially manufactured hand sanitizer and 71.4% indicated their hand hygiene adherence would improve with commercial hand sanitizer.

**Table 6:** Health care workers perceptions of alcohol-based hand sanitizers (N = 161)

**Attitude**	**Agree**	**Disagree**	**No opinion**
	**N (%)**		
**When using locally produced hand sanitizer**			
APPEARANCE: My hands appear red, blotchy, or have a rash	48 (29.8)	76 (47.2)	37 (23.0)
MOISTURE: The Skin on my hands becomes dry	76 (47.2)	59 (36.6)	26(16.1)
SENSATION: My hands itch or burn	30 (18.6)	98 (60.9)	33 (20.5)
INTACTNESS: There are abrasions or cracks on my hands	37 (23.0)	89 (55.3)	35 (21.7)
**When using commercial hand sanitizer**			
APPEARANCE: My hands appear red, blotchy, or have a rash	10 (6.2)	126 (78.3)	25 (15.5)
MOISTURE: The Skin on my hands becomes dry	21 (13.0)	110 (68.3)	30(18.6)
SENSATION: My hands itch or burn	7 (4.3)	131 (81.4)	23 (14.3)
INTACTNESS: There are abrasions or cracks on my hands	10 (6.2)	125 (77.6)	26 (16.1)
**Comparison: locally produced and commercial hand sanitizer**			
Commercial HS^a^ causes less skin irritation than locally produced HS	68 (42.2)	47 (29.2)	46 (28.6)
I prefer commercial HS over locally produced HS	103 (64.0)	19 (11.8)	39 (24.2)
I am more likely to clean my hands if I have commercial HS available	115 (71.4)	22 (13.7)	24 (14.9)

^a^HS, hand sanitizer.

## Discussion

This study demonstrates the effectiveness of the WHO Multimodal Hand Hygiene Strategy [1] in a resource-limited setting, on a short term basis. Implementation of the campaign was associated with a significant increase in HCW hand hygiene adherence from 2.1% at to 12.7% overall. While the post-intervention hand hygiene adherence rate remains sub-optimal, we believe that the significant increase in HCW hand hygiene adherence represents a substantial improvement that will serve as the basis for ongoing and future efforts at improving hand hygiene.

We documented extremely low baseline rates of hand hygiene adherence (2.1% overall). To our knowledge, this represents the lowest reported rate of hand hygiene adherence [1,11,20-22], but suspect that our findings are very typical of hand hygiene adherence rates in much, if not most, of Sub-Saharan Africa. While hand hygiene is beginning to attract more attention, infection control and prevention has not been emphasized or prioritized in most resource limited countries especially in Sub-Saharan Africa. Few such countries have well developed infection control and prevention programs [23]. Our hand hygiene campaign at a teaching hospital in Ethiopia represents only the second study to report the implementation of the WHO Multimodal Hand Hygiene Strategy in Sub-Saharan Africa [1,11]. The only other study in Sub-Saharan Africa was conducted in Mali, which showed similar increases in hand hygiene adherence (13.8% increase vs. our 10.6% increase) [11].

The lack of role models or hand hygiene champions, particularly among the physician group, was a behavioral factor that potentially contributed to poor hand hygiene adherence. We found that hand hygiene adherence significantly decreased when HCWs were on rounds with an attending (faculty) physician. Furthermore, attending physicians were the only group that did not significantly improve hand hygiene adherence rates following the intervention, despite having undergone the same training as all other HCWs. While numerous reports have demonstrated that physicians generally have the lowest rates of hand hygiene adherence among all HCW categories, there are little data showing that the presence of a poor role model can be detrimental to hand hygiene adherence [24]. This finding may be subject to confounding due to the number of people on attending rounds; in addition, there were limited number of observations that included the presence of faculty physicians. However, it is an area that warrants further investigation, as attending physicians play an integral role in infection prevention within the hospital setting.

The poor tolerability of the WHO-recommended formulation of hand sanitizer was an important finding of our study and was identified as a barrier to hand hygiene in our setting. A previous multicenter study demonstrated high rates of acceptability with the WHO formulation of hand sanitizer, although there were a few issues regarding the unpleasantness of smell, stickiness and skin break-down [25]. Survey of health care workers in our study found poor tolerability overall of the WHO-recommended formulation, as well as increased rates of reported skin dryness, irritation, and rashes with the use of WHO formulation sanitizer. Furthermore, our surveyed HCWs clearly preferred the commercially manufactured hand sanitizer. A large majority (71.4%) stated that having access to commercially prepared sanitizer would change their behavior and cause them to perform hand hygiene more frequently. It is important to note that these findings were the result of a perceptions survey, and therefore may be subject to a variety of bias. However, we believe that the perceptions of HCWs and their clear preference for commercial hand sanitizer requires further investigation as these findings may represent a previously unidentified barrier to hand hygiene adherence in Ethiopia and possibly has implications for other resource limited countries.

The sustainability of this hand hygiene initiative remains an ongoing challenge, due in large part to unsatisfactory options for a tolerable low-cost waterless hand sanitizer. In-house production of the WHO formulation hand sanitizer was determined to be a poor option for HCWs in our setting due to poor tolerability, despite its cost effectiveness. The continued importation of commercially produced hand sanitizer from the United States while well tolerated by HCWs, is neither logistically feasible nor cost effective and is not sustainable. One possible alternative is the local production of commercial hand sanitizer; at this time discussions are ongoing with a manufacturer in Ethiopia to explore the feasibility of producing a better-tolerated product in country. We suspect this is going to be a major issue in other resource-limited countries. Further data and investigation of this issue is urgently needed.

Our study was subject to several limitations. The first is that the study took place over a short duration (4 months overall including a six-week intervention period). This brief intervention differs from the WHO strategic approach, which recommends a minimum of 1 year for the intervention phase. This short duration potentially contributed to the limited impact on hand hygiene adherence demonstrated with this study. There was also close temporal proximity of the intervention and follow up observation period. While it has been shown that immediate advances can correlate with long term progress [26], it is possible that there was an immediate surge in hand hygiene adherence that may wane over time. Further follow up is needed to determine if this increase in adherence is being sustained. An additional limitation was the possibility of HCW recognition of the hand hygiene observer (especially in the post-intervention period) and subsequent change in their behavior. In our study, a single observer (KS) conducted all observations. While the observer’s purpose was not disclosed to the HCWs, it is possible that there was an increase in recognition of the observer over the duration of this study. We do not believe this accounted for the 6-fold increase in hand hygiene adherence in the post-intervention period but could have affected the behavior of some HCWs. The benefit of a single observer was to eliminate observer-to-observer variation in data collection and the benefits were thought to outweigh the potential for bias.

## Conclusion

This study represents a successful implementation of the WHO Multimodal Hand Hygiene Strategy at a teaching hospital in Ethiopia. Our study represents only the second report of implementation of the WHO Multimodal Hand Hygiene Strategy in Sub-Saharan African, and the first conducted in Ethiopia. This study adds to the paucity of data on hand hygiene initiatives in resource limited settings and lends credibly to the WHO-recommended methodology. Further work is needed to ensure sustainability of the gains made following implementation of the campaign and to continue efforts to improve adherence to hand hygiene by HCWs. Poor tolerability of the WHO-recommended formulation for hand sanitizer appeared to be a barrier to hand hygiene adherence in our study based on HCW feedback including the perception survey. Further research is needed to assess the tolerability of the WHO-recommended formulation for hand sanitizer.

## Abbreviations

HCW: Health care worker; HS: Hand sanitizer; OB/GYN: Obstetrics and gynecology; WHO: World Health Organization.

## Competing interests

All authors report no competing interests (financial or otherwise) relevant to this article.

## Author’s contributions

KS Study Design, Data Collection, Intervention Coordination, Data Analysis, Manuscript Creation. RK Study Design, Data Analysis, Manuscript Editing. AT Study Design, Intervention Coordination, Data Collection, Data Analysis, Manuscript Editing. ES Study Design, Data Analysis, Manuscript Editing. EA Study Design, Intervention Coordination, Data Collection. LT Study Design, Intervention Coordination, Manuscript Review. EKJ Study Design, Intervention Coordination, Data Collection. HMB Study design, Data Analysis, Manuscript Editing. All authors read and approved the final manuscript.

## Author’s information

All authors report no additional personal information relevant to this article.

## Supplementary Material

Additional file 1 Hand hygiene adherence observation form. Click here for file

Additional file 2 Hand hygiene poster. Click here for file

Additional file 3 Perceptions survey. Click here for file
