# Supplementary material for: Effectiveness of a multimodal hand hygiene campaign and obstacles to success in Addis Ababa, Ethiopia
Source: Antimicrob Resist Infect Control. 2014 Mar 17;3:8. doi: 10.1186/2047-2994-3-8 (PMC4004416; doi:10.1186/2047-2994-3-8)
Supplement: Additional file 1 — Hand hygiene adherence observation form. [file 2047-2994-3-8-S1.pdf]

Position Key: 1-Faculty 2-Resident 3-Student 4-Nurse 5-Other HCW 6-unknown

Date:				
Location:				
#	Position	Indication	Action	Rounds
1		☐Before ☐After	☐HW ☐HR ☐None	☐Y ☐N
2		☐Before ☐After	☐HW ☐HR ☐None	☐Y ☐N
3		☐Before ☐After	☐HW ☐HR ☐None	☐Y ☐N
4		☐Before ☐After	☐HW ☐HR ☐None	☐Y ☐N
5		☐Before ☐After	☐HW ☐HR ☐None	☐Y ☐N
6		☐Before ☐After	☐HW ☐HR ☐None	☐Y ☐N
7		☐Before ☐After	☐HW ☐HR ☐None	☐Y ☐N
8		☐Before ☐After	☐HW ☐HR ☐None	☐Y ☐N
9		☐Before ☐After	☐HW ☐HR ☐None	☐Y ☐N
10		☐Before ☐After	☐HW ☐HR ☐None	☐Y ☐N
11		☐Before ☐After	☐HW ☐HR ☐None	☐Y ☐N
12		☐Before ☐After	☐HW ☐HR ☐None	☐Y ☐N
13		☐Before ☐After	☐HW ☐HR ☐None	☐Y ☐N
14		☐Before ☐After	☐HW ☐HR ☐None	☐Y ☐N
15		☐Before ☐After	☐HW ☐HR ☐None	☐Y ☐N
16		☐Before ☐After	☐HW ☐HR ☐None	☐Y ☐N
17		☐Before ☐After	☐HW ☐HR ☐None	☐Y ☐N
18		☐Before ☐After	☐HW ☐HR ☐None	☐Y ☐N
19		☐Before ☐After	☐HW ☐HR ☐None	☐Y ☐N
20		☐Before ☐After	☐HW ☐HR ☐None	☐Y ☐N
21		☐Before ☐After	☐HW ☐HR ☐None	☐Y ☐N
22		☐Before ☐After	☐HW ☐HR ☐None	☐Y ☐N
23		☐Before ☐After	☐HW ☐HR ☐None	☐Y ☐N
24		☐Before ☐After	☐HW ☐HR ☐None	☐Y ☐N
25		☐Before ☐After	☐HW ☐HR ☐None	☐Y ☐N

Position Key: 1-Faculty 2-Resident 3-Student 4-Nurse 5-Other HCW 6-unknown

Date:				
Location:				
#	Position	Indication	Action	Rounds
26		☐Before ☐After	☐HW ☐HR ☐None	☐Y ☐N
27		☐Before ☐After	☐HW ☐HR ☐None	☐Y ☐N
28		☐Before ☐After	☐HW ☐HR ☐None	☐Y ☐N
29		☐Before ☐After	☐HW ☐HR ☐None	☐Y ☐N
30		☐Before ☐After	☐HW ☐HR ☐None	☐Y ☐N
31		☐Before ☐After	☐HW ☐HR ☐None	☐Y ☐N
32		☐Before ☐After	☐HW ☐HR ☐None	☐Y ☐N
33		☐Before ☐After	☐HW ☐HR ☐None	☐Y ☐N
34		☐Before ☐After	☐HW ☐HR ☐None	☐Y ☐N
35		☐Before ☐After	☐HW ☐HR ☐None	☐Y ☐N
36		☐Before ☐After	☐HW ☐HR ☐None	☐Y ☐N
37		☐Before ☐After	☐HW ☐HR ☐None	☐Y ☐N
38		☐Before ☐After	☐HW ☐HR ☐None	☐Y ☐N
39		☐Before ☐After	☐HW ☐HR ☐None	☐Y ☐N
40		☐Before ☐After	☐HW ☐HR ☐None	☐Y ☐N
41		☐Before ☐After	☐HW ☐HR ☐None	☐Y ☐N
42		☐Before ☐After	☐HW ☐HR ☐None	☐Y ☐N
43		☐Before ☐After	☐HW ☐HR ☐None	☐Y ☐N
44		☐Before ☐After	☐HW ☐HR ☐None	☐Y ☐N
45		☐Before ☐After	☐HW ☐HR ☐None	☐Y ☐N
46		☐Before ☐After	☐HW ☐HR ☐None	☐Y ☐N
47		☐Before ☐After	☐HW ☐HR ☐None	☐Y ☐N
48		☐Before ☐After	☐HW ☐HR ☐None	☐Y ☐N
49		☐Before ☐After	☐HW ☐HR ☐None	☐Y ☐N
50		☐Before ☐After	☐HW ☐HR ☐None	☐Y ☐N
