# Supplementary material for: Effectiveness of a multimodal hand hygiene campaign and obstacles to success in Addis Ababa, Ethiopia
Source: Antimicrob Resist Infect Control. 2014 Mar 17;3:8. doi: 10.1186/2047-2994-3-8 (PMC4004416; doi:10.1186/2047-2994-3-8)
Supplement: Additional file 2 — Hand hygiene poster. [file 2047-2994-3-8-S2.pdf]

Have YOU Cleaned Your Hands?

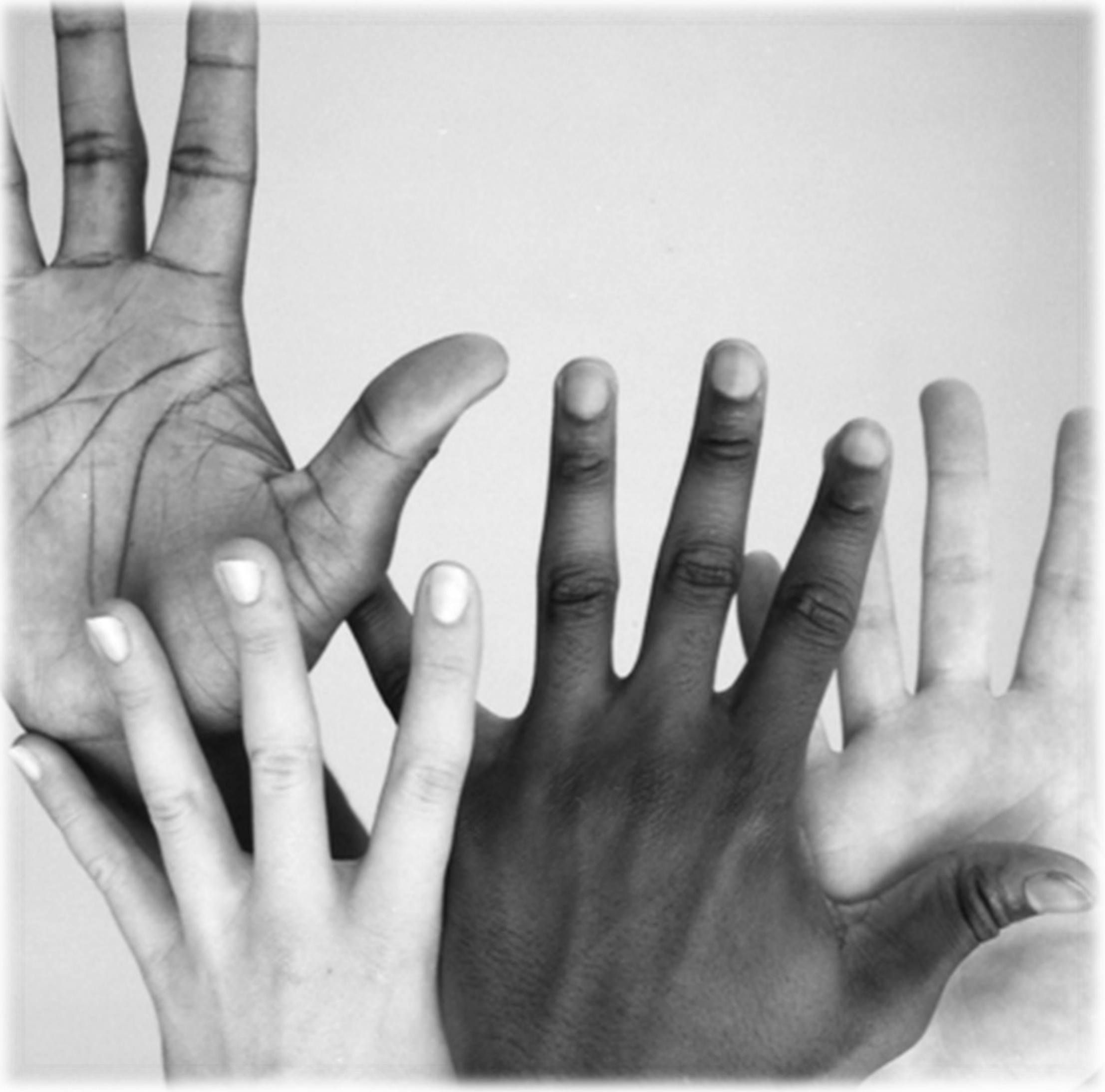

Hand Hygiene Saves Lives
