# Supplementary material for: Effectiveness of a multimodal hand hygiene campaign and obstacles to success in Addis Ababa, Ethiopia
Source: Antimicrob Resist Infect Control. 2014 Mar 17;3:8. doi: 10.1186/2047-2994-3-8 (PMC4004416; doi:10.1186/2047-2994-3-8)
Supplement: Additional file 3 — Perceptions survey. [file 2047-2994-3-8-S3.pdf]

Thank you for taking the time to complete this survey about hand hygiene. This is a completely anonymous survey, so please respond to each question as accurately and honestly as possible.

1. Over the past 3 months, have you received training about the importance of hand hygiene?

- ☐ Yes
- ☐ No
- ☐ Do not know/unsure

	Not at all	Little	Somewhat	A Lot	A Great Deal
2. To what extent did training improve your knowledge of the importance of hand hygiene for infection control?	1	2	3	4	5
3. To what extent did training improve your understanding of when and how to perform hand hygiene?	1	2	3	4	5
4. To what extent did training increase your frequency of performing hand hygiene?	1	2	3	4	5

5. Do you have hand sanitizer in your possession at this time?

- ☐ Yes
- ☐ No

6. What difficulties have you faced in getting access to commercial sanitizer (Purell) over the past 2 months? (Select all that apply)

- ☐ Did not receive initial bottle of commercial (Purell) hand sanitizer
- ☐ Unable to get refill of commercial (Purell) hand sanitizer
- ☐ Lost original bottle of commercial (Purell) hand sanitizer
- ☐ No difficulties
- ☐ Other (please specify):________________________________

	Never	Rarely	Sometimes	Often	Always
7. How often do you clean your hands *prior* to eating a meal?	1	2	3	4	5
8. How often do you clean your hands *after* eating a meal?	1	2	3	4	5
9. How often do you clean your hands *after* toileting?	1	2	3	4	5
10. How often do you clean your hands *prior* to patient contact?	1	2	3	4	5
11. How often do you clean your hands *after* patient contact?	1	2	3	4	5

When Using Locally Produced (Purple) Sanitizer	Strongly Disagree	Disagree	Neutral	Agree	Strongly Agree
12. APPEARANCE: My hand appear red, blotchy, or have a rash	1	2	3	4	5
13. MOISTURE: The skin on my hands becomes dry	1	2	3	4	5
14. SENSATION: My hands itch or burn	1	2	3	4	5
15. INTACTNESS: There are abrasions or cracks on my hands	1	2	3	4	5

When Using Commercial Sanitizer (Purell)	Strongly Disagree	Disagree	Neutral	Agree	Strongly Agree
16. APPEARANCE: My hand appear red, blotchy, or have a rash	1	2	3	4	5
17. MOISTURE: The skin on my hands becomes dry	1	2	3	4	5
18. SENSATION: My hands itch or burn	1	2	3	4	5
19. INTACTNESS: There are abrasions or cracks on my hands	1	2	3	4	5

COMPARISON: Locally Produced and Commercial Sanitizer	Strongly Disagree	Disagree	Neutral	Agree	Strongly Agree
20. Commercial (Purell) hand sanitizer causes less skin irritation than the locally produced (purple) hand sanitizer	1	2	3	4	5
21. I prefer the commercial (Purell) hand sanitizer over the locally produced (purple) hand sanitizer	1	2	3	4	5
22. I am more likely to clean my hands if I have commercial (Purell) hand sanitizer available.	1	2	3	4	5

---

23. How old are you (in years)? _________ years old

24. What is your gender?

- ☐ Male
- ☐ Female

25. What is your primary job title? (Check only one):

- ☐ Faculty physician
- ☐ Resident physician
- ☐ Medical Student/Intern
- ☐ Nurse
- ☐ Other (please specify): _______________

26. What is your primary department? (Check only one):

- ☐ Medicine
- ☐ Surgery
- ☐ GYN/OBS
- ☐ Pediatrics
- ☐ Emergency
- ☐ Other (please specify): ________________
